# Supplementary material for: ALPK1 hotspot mutation as a driver of human spiradenoma and spiradenocarcinoma
Source: Nat Commun. 2019 May 17;10:2213. doi: 10.1038/s41467-019-09979-0 (PMC6525246; doi:10.1038/s41467-019-09979-0)
Supplement: Supplementary file 1 — Supplementary Information [file 41467_2019_9979_MOESM1_ESM.pdf]

Supplementary Information

ALPK1 hotspot mutation as a driver of human spiradenoma and spiradenocarcinoma

Adams et al.

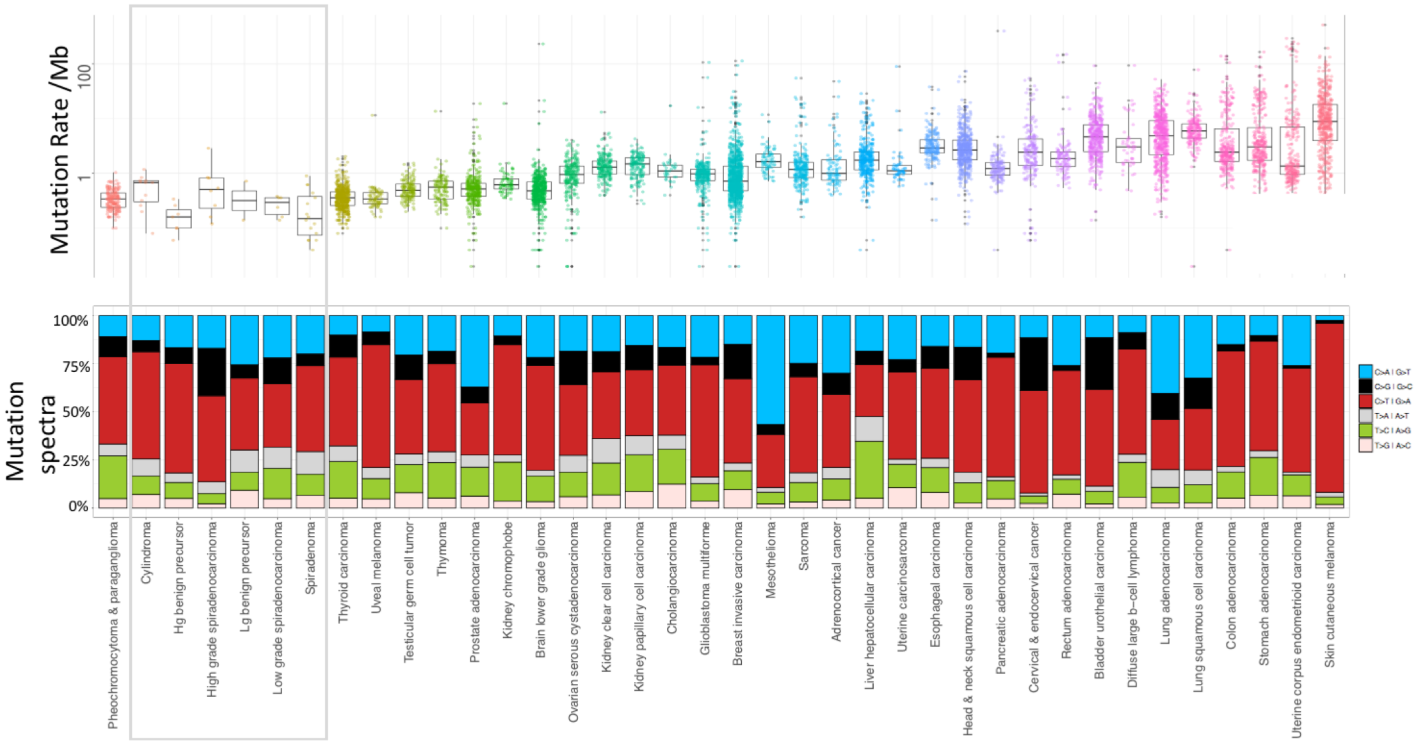

Supplementary Figure 1: The mutation rate of the adnexal tumors analysed in this study compared to TCGA tumour samples. Upper panel shows mutation rate while the bottom panel represents mutation spectra across the different cancer types. Mutation rate and spectra were calculated using exonic mutations. Cylindroma, Spiradenoma, malignant low and high-grade Spiradenocarcinoma and their benign regions are shown inside the grey rectangle.

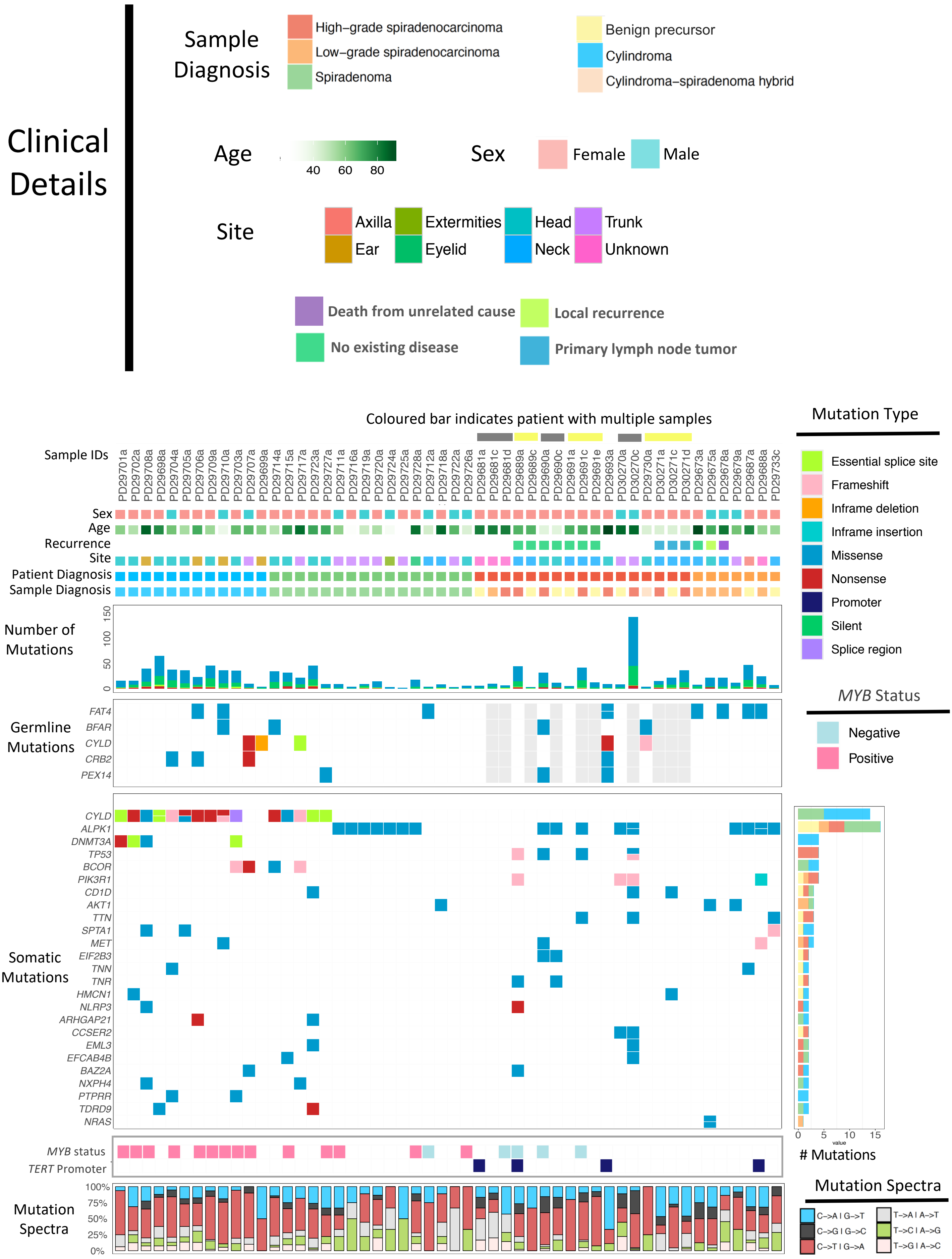

Supplementary Figure 2: Profile of mutated genes and clinical data.

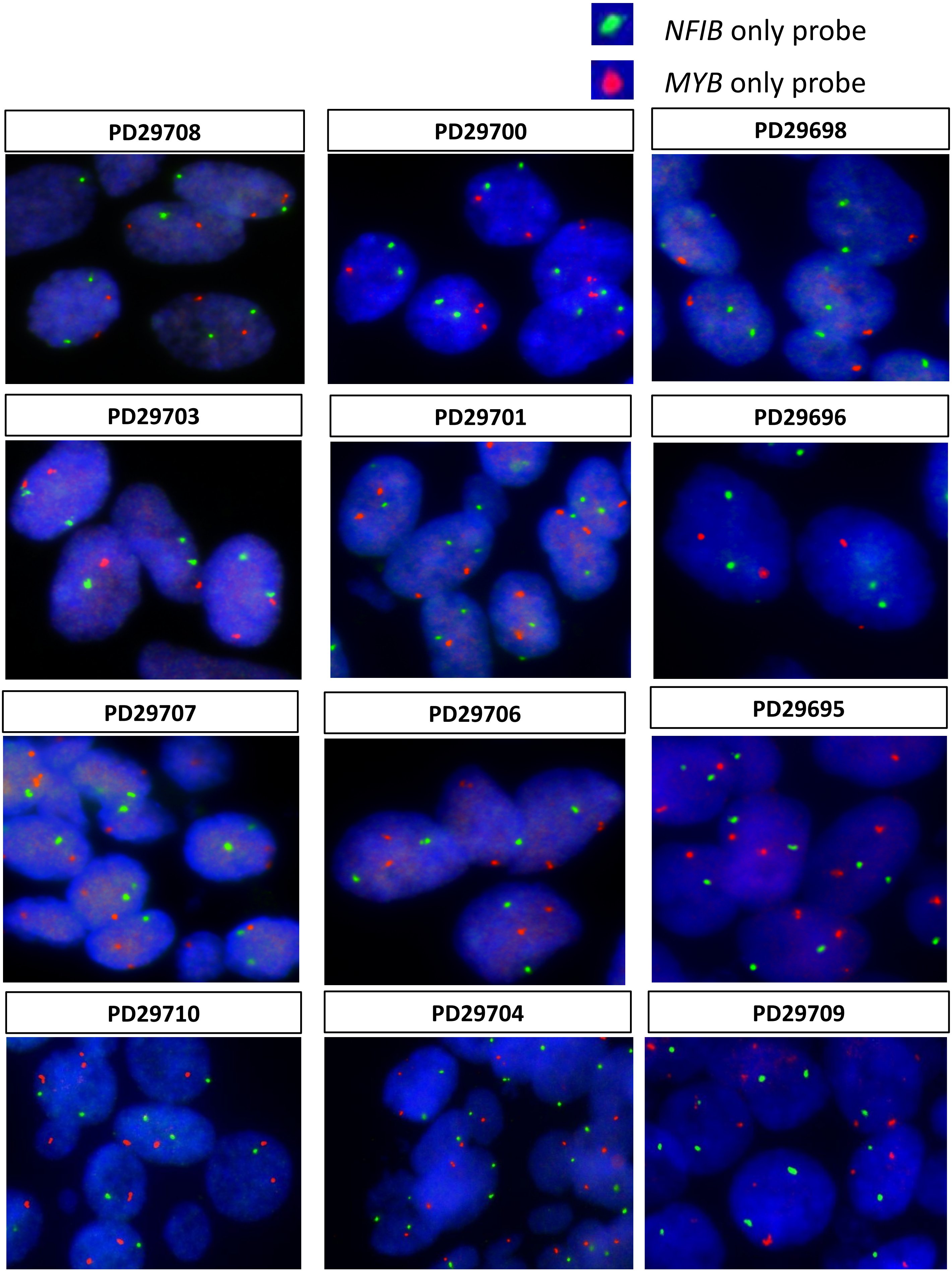

Supplementary Figure 3 : Fluorescence *in situ* hybridization (FISH) for the *MYB-NFIB* fusion in cylindromas. Representative images for each case are shown. See Methods and Supplementary Table 1 for further details.

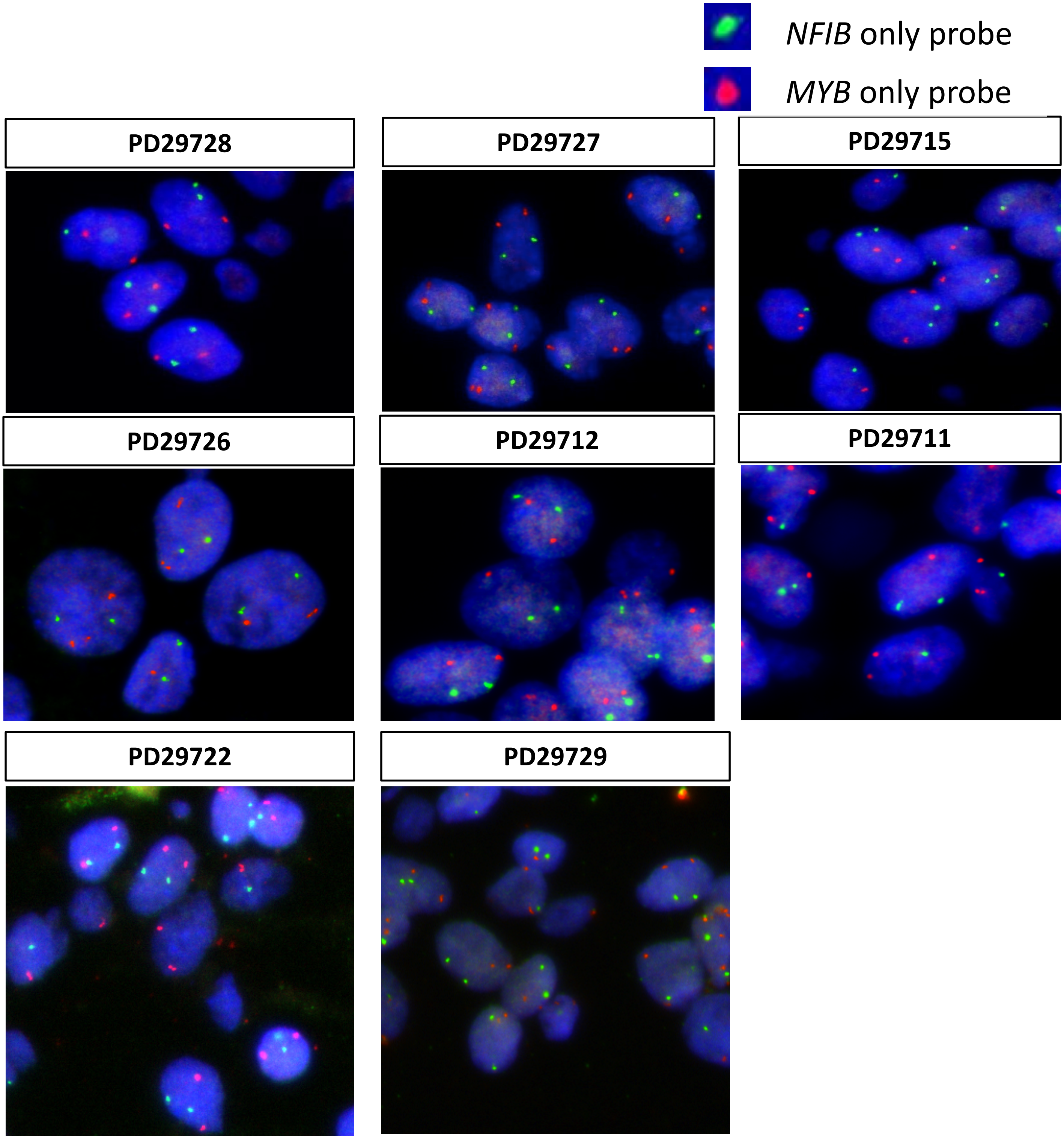

Supplementary Figure 4 : Fluorescence *in situ* hybridization (FISH) for the *MYB-NFIB* fusion in spiradenoma samples (PD29728, PD29727, PD29715, PD29726, PD29712, PD29711, PD29722) and a cylindroma-Spiradenoma hybrid tumor (PD29729). See Methods and Supplementary Table 1 for further details.

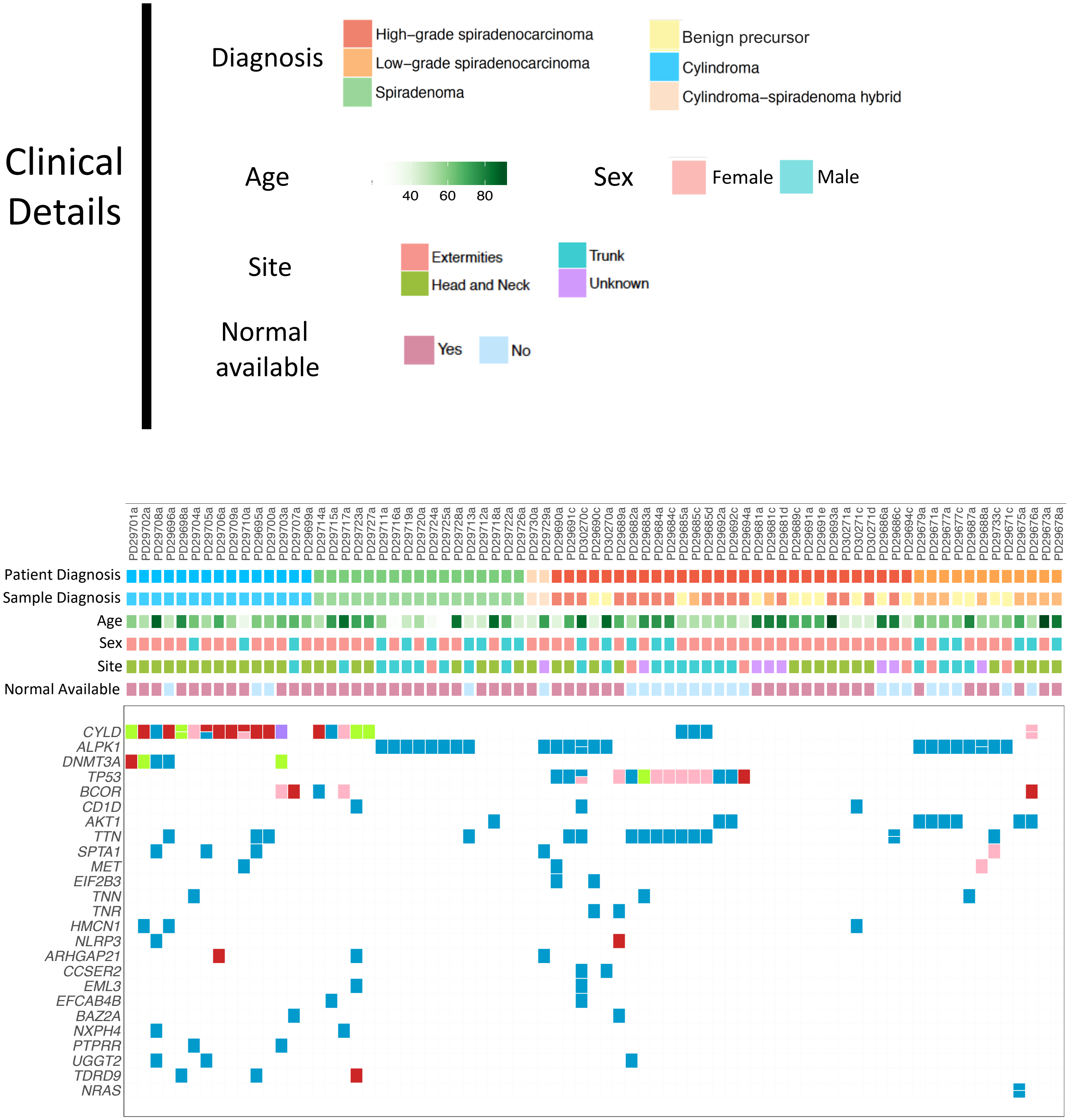

Supplementary Figure 5 : Driver gene mutation landscape of all 75 samples/tumours. See Supplementary Table 1 for sample details. Mutation types are the same as shown in Figure 1 and Supplementary Figure 2.

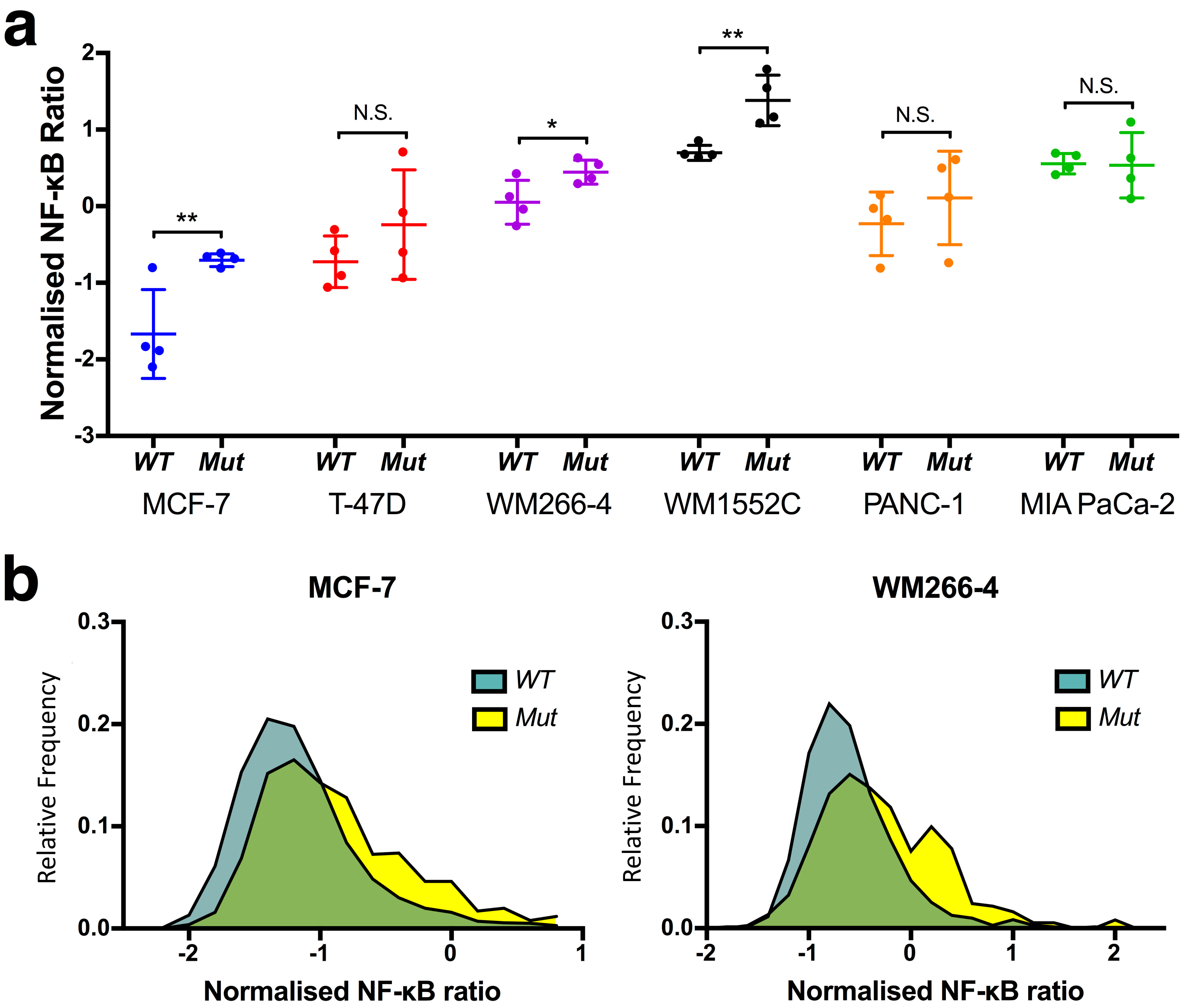

Supplementary Figure 6 : Effects of the p.V1092A *ALPK1* mutation on NF-kB nuclear translocation in cancer cell lines. Cells were transfected with a wildtype or an *ALPK1* p.V1092A cDNA construct together with a red fluorescent protein (RFP) construct and p65/NF-kB ratios (nuclear to cytoplasmic intensity) were measured in RFP-positive cells (see Methods). Each data point is the mean of 6 technical replicates (each 500 RFP-positive cells) and the experiment was replicated four times (independent biological replicates). Y Axis is a log scale. **a,** NF-kB ratios for each cell line. N.S. = non-significant, *$P < 0.05$, **$P = 0.01$, one-tailed t-test; bars, standard deviation. **b,** Frequency distributions of NF-kB ratios in MCF-7 and WM266-4 cells.

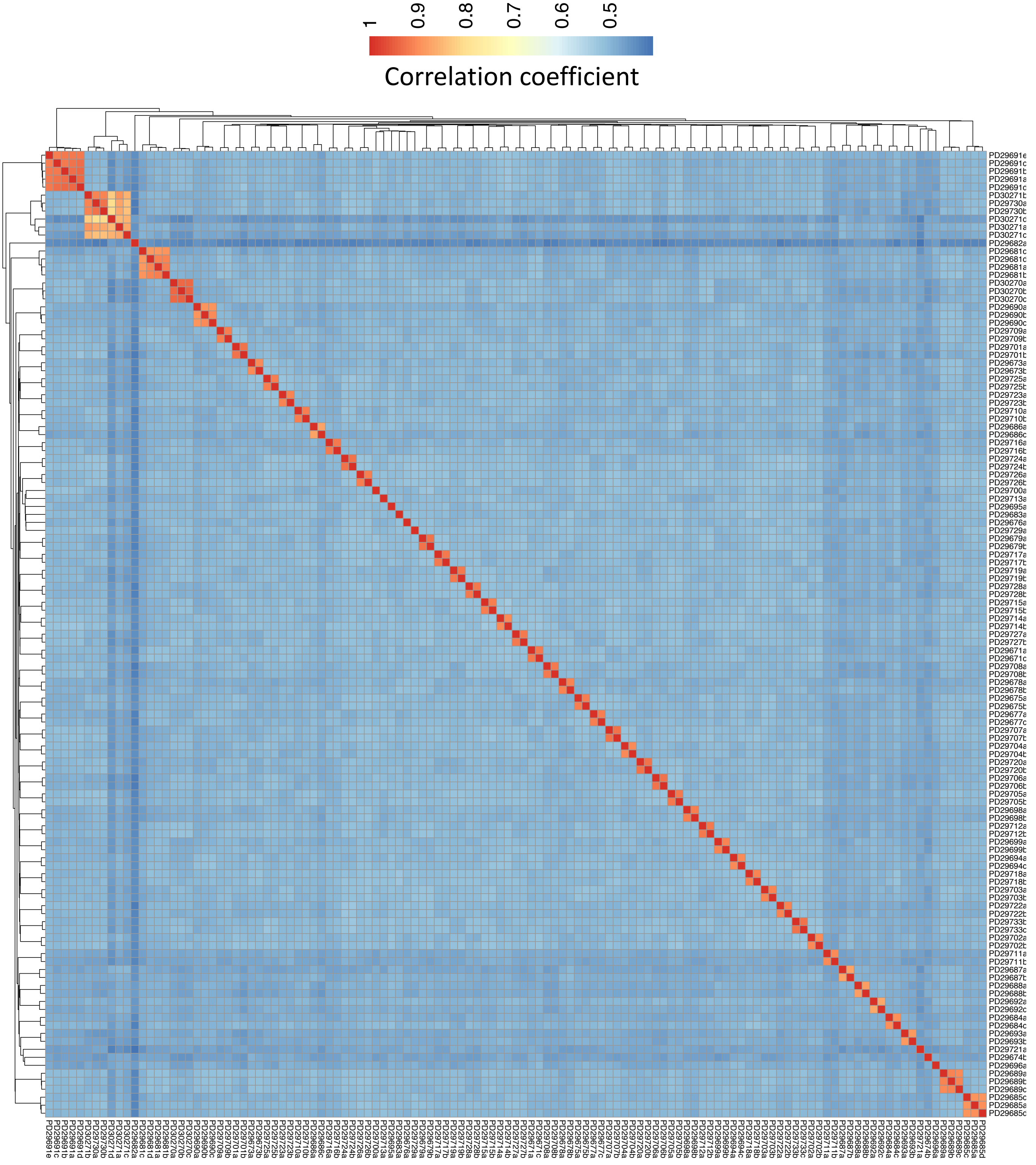

Supplementary Figure 7 : Sample swap assessment with genotype based correlation. 20,000 germline SNP positions were used to assess for similarity measure. Samples originated from the same patient (tumour, normal, benign precursors) shows strongest correlation among themselves.

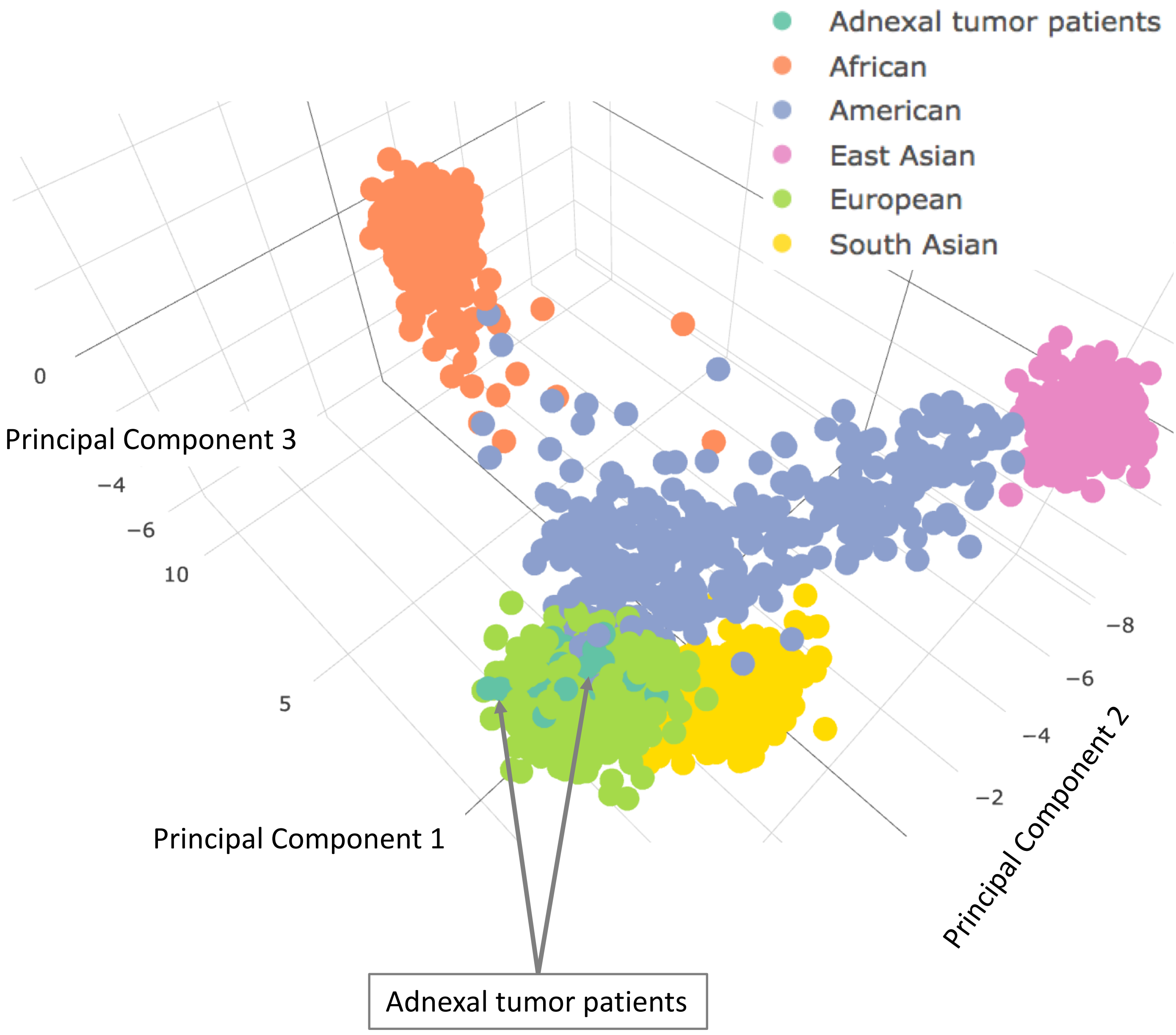

Supplementary Figure 8 : Principal component analysis (PCA) using 1000 genome population data to identify the ethnicity of patients in our skin adnexal tumour cohort. Three dimensional (3D) representation of the first three principal components demonstrating that all of our adnexal patients were of European descent.
