# Supplementary material for: ALPK1 hotspot mutation as a driver of human spiradenoma and spiradenocarcinoma
Source: Nat Commun. 2019 May 17;10:2213. doi: 10.1038/s41467-019-09979-0 (PMC6525246; doi:10.1038/s41467-019-09979-0)
Supplement: Supplementary file 3 — Description of Additional Supplementary Files [file 41467_2019_9979_MOESM3_ESM.pdf]

Description of Additional Supplementary Information

File Name: Supplementary Data 1
Description: Samples and summary of the analysis

File Name: Supplementary Data 2
Description: Summary of the variants identified in this study

File Name: Supplementary Data 3
Description: statistical analysis of driver genes

File Name: Supplementary Data 4
Description: MYB IHC

File Name: Supplementary Data 5
Description: Genes in targeted gene panel
