# Supplementary material for: ALPK1 hotspot mutation as a driver of human spiradenoma and spiradenocarcinoma
Source: Nat Commun. 2019 May 17;10:2213. doi: 10.1038/s41467-019-09979-0 (PMC6525246; doi:10.1038/s41467-019-09979-0)
Supplement: Supplementary file 9 — Reporting Summary [file 41467_2019_9979_MOESM9_ESM.pdf]

nature research

Corresponding author(s): NCOMMS-18-35084-T
David Adams

Last updated by author(s): 17/03/19

Reporting Summary

Nature Research wishes to improve the reproducibility of the work that we publish. This form provides structure for consistency and transparency in reporting. For further information on Nature Research policies, see Authors & Referees and the Editorial Policy Checklist.

Statistics

For all statistical analyses, confirm that the following items are present in the figure legend, table legend, main text, or Methods section.

n/a	Confirmed	
☐	☒	The exact sample size (*n*) for each experimental group/condition, given as a discrete number and unit of measurement
☐	☒	A statement on whether measurements were taken from distinct samples or whether the same sample was measured repeatedly
☐	☒	The statistical test(s) used AND whether they are one- or two-sided<br>*Only common tests should be described solely by name; describe more complex techniques in the Methods section.*
☐	☒	A description of all covariates tested
☐	☒	A description of any assumptions or corrections, such as tests of normality and adjustment for multiple comparisons
☐	☒	A full description of the statistical parameters including central tendency (e.g. means) or other basic estimates (e.g. regression coefficient) AND variation (e.g. standard deviation) or associated estimates of uncertainty (e.g. confidence intervals)
☐	☒	For null hypothesis testing, the test statistic (e.g. *F*, *t*, *r*) with confidence intervals, effect sizes, degrees of freedom and *P* value noted<br>*Give P values as exact values whenever suitable.*
☒	☐	For Bayesian analysis, information on the choice of priors and Markov chain Monte Carlo settings
☒	☐	For hierarchical and complex designs, identification of the appropriate level for tests and full reporting of outcomes
☒	☐	Estimates of effect sizes (e.g. Cohen's *d*, Pearson's *r*), indicating how they were calculated

Our web collection on statistics for biologists contains articles on many of the points above.

Software and code

Policy information about availability of computer code

Data collection	All of the software used and the version numbers are stated in the manuscript. Briefly:<br>BioBamBam (v2.0.54)<br>BWA (v0.7.12)<br>CaVEMan/cgppindel (v0.2.4w)<br>DISCOVER<br>deconstructSigs<br>Sequenza software package (v2.1.0)
Data analysis	We didn't write any code specifically for this project. We used the packages above.

For manuscripts utilizing custom algorithms or software that are central to the research but not yet described in published literature, software must be made available to editors/reviewers. We strongly encourage code deposition in a community repository (e.g. GitHub). See the Nature Research guidelines for submitting code & software for further information.

Data

Policy information about availability of data

All manuscripts must include a data availability statement. This statement should provide the following information, where applicable:

- Accession codes, unique identifiers, or web links for publicly available datasets
- A list of figures that have associated raw data
- A description of any restrictions on data availability

The data has been accessioned under the study EGAS00001001799 in the European Genome-phenome Archive. Source Data File 1 provides all of the variant calls in MAF and VCF format.

Field-specific reporting

Please select the one below that is the best fit for your research. If you are not sure, read the appropriate sections before making your selection.

☒ Life sciences ☐ Behavioural & social sciences ☐ Ecological, evolutionary & environmental sciences

For a reference copy of the document with all sections, see nature.com/documents/nr-reporting-summary-flat.pdf

Life sciences study design

All studies must disclose on these points even when the disclosure is negative.

Sample size	We sequenced all of the tumours we could obtain.
Data exclusions	No samples were excluded from the analysis in the paper. A few of the samples we obtained from our collaborators were either of poor quality or did not sequence well and thus we did not include these in our analysis.
Replication	The functional experiment was repeated three times.
Randomization	N/A
Blinding	N/A

Reporting for specific materials, systems and methods

We require information from authors about some types of materials, experimental systems and methods used in many studies. Here, indicate whether each material, system or method listed is relevant to your study. If you are not sure if a list item applies to your research, read the appropriate section before selecting a response.

Materials & experimental systems

n/a	Involved in the study	
☐	☒	Antibodies
☐	☒	Eukaryotic cell lines
☒	☐	Palaeontology
☒	☐	Animals and other organisms
☐	☒	Human research participants
☐	☒	Clinical data

Methods

n/a	Involved in the study	
☒	☐	ChIP-seq
☒	☐	Flow cytometry
☒	☐	MRI-based neuroimaging

Antibodies

Antibodies used	anti-MYB monoclonal antibody (1:200 dilution; clone EP769Y; Abcam, Cambridge, MA, USA)<br>anti-NF-κB p65 antibody (1:5000 dilution; clone D14E12; Cell Signaling Technology, Danvers, MA, USA)
Validation	Both of these antibodies are used clinically. Thus are well tested. Further, results were assessed by pathologists to score for characteristic staining such as in immune cells as an indicator of staining success.

Eukaryotic cell lines

Policy information about cell lines

Cell line source(s)	All cell lines were from ATCC
Authentication	All lines were STR profiled to confirm their identity.
Mycoplasma contamination	Cell lines used were from mycoplasma-free stocks
Commonly misidentified lines (See ICLAC register)	This database was searched on the 24th Feb and none of the cell lines were found.

Human research participants

Policy information about studies involving human research participants

Population characteristics	N/A
Recruitment	All cases we could obtain were included.
Ethics oversight	All samples were obtained through the West Lothian Tissue bank with REC 15/ES/0094. The project was approved by the Sanger Human Materials and Data Management Committee.

Note that full information on the approval of the study protocol must also be provided in the manuscript.

Clinical data

Policy information about clinical studies

All manuscripts should comply with the ICMJE guidelines for publication of clinical research and a completed CONSORT checklist must be included with all submissions.

Clinical trial registration	N/A
Study protocol	N/A
Data collection	Clinical data was obtained from the collaborating pathologists.
Outcomes	*Describe how you pre-defined primary and secondary outcome measures and how you assessed these measures.*
